# Change in glomerular filtration rate over time in the Oxford Renal Cohort Study: observational study

**DOI:** 10.3399/BJGP.2021.0477

**Published:** 2022-02-22

**Authors:** Jennifer A Hirst, Maarten W Taal, Simon DS Fraser, José M Ordóñez Mena, Chris A O’Callaghan, Richard J McManus, Clare J Taylor, Yaling Yang, Emma Ogburn, FD Richard Hobbs

**Affiliations:** Nuffield Department of Primary Care Health Sciences, University of Oxford, Oxford; National Institute for Health Research (NIHR) Oxford Biomedical Research Centre, Oxford University Hospitals NHS Foundation Trust, Oxford.; Centre for Kidney Research and Innovation, Academic Unit for Translational Medical Sciences, School of Medicine, University of Nottingham, Nottingham; honorary consultant nephrologist, University Hospitals of Derby and Burton NHS Foundation Trust.; School of Primary Care, Population Sciences and Medical Education, Faculty of Medicine, University of Southampton, Southampton General Hospital, Southampton.; Nuffield Department of Primary Care Health Sciences, University of Oxford, Oxford; NIHR Oxford Biomedical Research Centre, Oxford University Hospitals NHS Foundation Trust, Oxford.; NIHR Oxford Biomedical Research Centre, Oxford University Hospitals NHS Foundation Trust, Oxford; Nuffield Department of Medicine, University of Oxford, Oxford.; Nuffield Department of Primary Care Health Sciences, University of Oxford, Oxford.; Nuffield Department of Primary Care Health Sciences, University of Oxford, Oxford; NIHR Oxford Biomedical Research Centre, Oxford University Hospitals NHS Foundation Trust, Oxford.; Nuffield Department of Primary Care Health Sciences, University of Oxford, Oxford; NIHR Oxford Biomedical Research Centre, Oxford University Hospitals NHS Foundation Trust, Oxford.; Nuffield Department of Primary Care Health Sciences, University of Oxford, Oxford.; Nuffield professor, head of primary care health sciences, Nuffield Department of Primary Care Health Sciences, University of Oxford, Oxford; provice chancellor, director, NIHR Applied Research Collaboration Oxford Biomedical Research Centre, Oxford University Hospitals NHS Foundation Trust, Oxford.

**Keywords:** chronic kidney diseases, cohort studies, glomerular filtration rate, primary care

## Abstract

**Background:**

Decline in kidney function can result in adverse health outcomes. The Oxford Renal Cohort Study has detailed baseline assessments from 884 participants ≥60 years of age.

**Aim:**

To determine the proportion of participants with a decline in estimated glomerular filtration rate (eGFR), identify determinants of decline, and determine proportions with chronic kidney disease (CKD) remission.

**Design and setting:**

Observational cohort study in UK primary care.

**Method:**

Data were used from baseline and annual follow-up assessments to monitor change in kidney function. Rapid eGFR decline was defined as eGFR decrease >5 ml/min/1.73 m^2^/year, improvement as eGFR increase >5 ml/min/1.73 m^2^/year, and remission in those with CKD at baseline and eGFR >60 ml/min/1.73 m^2^ during follow-up. Cox proportional hazard models were used to identify factors associated with eGFR decline.

**Results:**

There was a net decline in eGFR in the 884 participants over 5 years of follow-up. In 686 participants with >2 eGFR tests with a median follow-up of 2.1 years, 164 (24%) evidenced rapid GFR decline, 185 (27%) experienced eGFR improvement, and 82 of 394 (21%) meeting CKD stage 1–4 at baseline experienced remission. In the multivariable analysis, smoking status, higher systolic blood pressure, and being known to have CKD at cohort entry were associated with rapid GFR decline. Those with CKD stage 3 at baseline were less likely to exhibit GFR decline compared with normal kidney function.

**Conclusion:**

This study established that 24% of people evidenced rapid GFR decline whereas 21% evidenced remission of CKD. People at risk of rapid GFR decline may benefit from closer monitoring and appropriate treatment to minimise risks of adverse outcomes, although only a small proportion meet the National Institute for Health and Care Excellence criteria for referral to secondary care.

## BACKGROUND

Chronic kidney disease (CKD) is defined as decreased estimated glomerular filtration rate (eGFR) or markers of renal damage of at least 3 months’ duration.^1^ Early stages of CKD are asymptomatic, but over time eGFR may decline and increase risk of cardiovascular disease,^2^^,^^3^ kidney failure,^4^ and premature mortality.^2^^,^^5^ A sustained decline in eGFR of >5 ml/min/year is the accepted definition of rapid decline in eGFR^1^ and factors associated with a decline in eGFR and progression of CKD include poor blood pressure control, diabetes,^6^ and obesity.^7^ Variability in eGFR itself is also associated with poor health outcomes including end-stage kidney disease,^8^ cardiovascular events,^9^ and mortality,^10^ and may therefore indicate reduced kidney resilience. Data from UK primary care indicate that many people with a diagnosis of CKD experience ‘remission’ of their CKD as they move back and forth across the 60 ml/min/1.73 m^2^ eGFR threshold over time.^11^

The prevalence of CKD increases with age^12^^,^^13^ and many people live with undiagnosed CKD.^14^^,^^15^ It is not well understood whether people with unidentified CKD are at similar risk of adverse health outcomes to those known to have CKD. The Oxford Renal Cohort Study (OxRen) was established in 2013 to screen an older population in UK primary care for all stages of CKD.^16^ The baseline characteristics of the study established that 56% of people with confirmed CKD were already known to have the condition at recruitment to the study, whereas 44% were diagnosed through screening.^15^ Some OxRen participants now have over 5 years’ follow-up data, allowing exploration of the changes in eGFR over time and identification of risk factors associated with eGFR decline. Follow-up data were used to determine the proportion of participants who experienced rapid eGFR decline, describe numbers of people moving in and out of remission, and identify determinants of rapid GFR decline and remission.

**Table:** How this fits in

The prevalence of chronic kidney disease (CKD) increases with age and some people are unaware that they have CKD. It is established that some people with CKD will go on to develop adverse health outcomes, and understanding what makes this more (or less) likely is important in primary care. This study reports the number of people with CKD who experience a rapid deterioration of kidney function measured as a decline in estimated glomerular filtration rate. It identifies factors associated with a rapid decline in kidney function and describes the number of people who enter and leave CKD remission during follow-up.

## METHOD

### Study population and laboratory methods

Participants aged ≥60 years in UK primary care were screened for CKD by determining their albumin–creatinine ratio (ACR) and Modification of Diet in Renal Disease eGFR.^17^ Briefly, participants were screened to identify those who had a CKD diagnosis confirmed by two positive tests a minimum of 90 days apart and those who did not have a CKD diagnosis, but had a single test with either a decreased eGFR (<60 ml/min/1.73 m^2^) or a raised urinary ACR (>3 mg/mmol). Participants with existing CKD or those with a positive screening test based on eGFR and ACR were invited to a baseline assessment. All laboratory measurements were performed in a single laboratory and those with existing CKD or a positive screening test based on eGFR and ACR attended a baseline assessment. Those who had a baseline assessment by December 2019 (*n* = 884) were included in the analysis. Full details of OxRen have been reported elsewhere.^15^ Data management decisions on laboratory test results made before analysis are detailed in the Supplementary data (see Supplementary Box S1).

### Outcomes

Potential CKD progression was calculated in participants who had ≥2 eGFR measurements at least 12 weeks apart.^18^ In the primary analysis, rapid GFR decline was defined as a decrease in eGFR of at least 5 ml/min/1.73 m^2^ per year in line with the Kidney Disease Improving Global Outcomes (KDIGO) definition.^1^ Numbers who met different definitions of CKD progression were also reported, including a decline in eGFR of at least 15 ml/min/1.73 m^2^ per year, a 25% decline in eGFR combined with progression to the next CKD stage (indications for referral to a nephrology service),^1^ and a decline in the slope of at least 5 ml/min/1.73 m^2^ per year through regression of ≥3 readings.

Improvement was defined in this study as both an increase in eGFR of at least 5 ml/min/1.73 m^2^ per year and remission as eGFR >60 ml/min/1.73 m^2^ and ACR <3 mg/mmol.^11^ Progression and remission of CKD in 403 people with full baseline and 2-year follow-up results were presented in a flow chart to show the proportions that enter and leave remission at each visit. Movement between each stage of CKD at baseline and at year 2 were presented in a table.

### Statistical analysis

All analyses were performed using Stata SE16 (Statacorp). Descriptive data on eGFR and numbers who progressed were presented in tables, bar charts, and graphs as mean and standard deviation (SD) of eGFR at baseline and each year of follow-up. Covariates used in the primary analysis included demographic data, anthropometric measurements, and history of CKD (see Supplementary Table S1). An extended analysis was conducted that included history of comorbidities collected at participant’s baseline assessment.

The primary analysis was conducted using univariable and multivariable Cox proportional hazards regression analyses to estimate hazard ratio (HR) and 95% confidence intervals (95% CIs) for the association of covariates with time to progression or censoring at the end of follow-up (December 2019). The dependent variable was time to first GFR that was >5 ml/min/1.73 m^2^ per year lower than the baseline value. The index date was the date of the participant’s baseline eGFR test. Assumption of proportional hazards was verified and no violations were observed for any of the predictors. Because of skewed data, urinary ACR was logarithmically transformed and alcohol consumption was dichotomised to no alcohol use or any alcohol use before analysis. Improvement in eGFR was analysed using the same covariates using Cox regression. All covariates were included in the multivariable analysis.

To determine whether eGFR variability was associated with rapid eGFR decline, participants’ laboratory eGFR results before and up to their baseline assessment date were used to calculate variability. Variability independent of the mean (VIM)^9^^,^^19^^,^^20^ was calculated using the following equation: 100 × SD/mean^β^. Where β was the regression coefficient, calculated from a simple linear regression of the natural logarithm of the SD against the natural logarithm of the mean within-individual eGFR.

The VIM was classified into four quartiles, the highest quartile representing the participants with the highest eGFR variability,^9^ which were used as categorical variables in the analysis. Because this method has not been widely used, sensitivity analyses were performed using mean eGFR and SD,^10^^,^^21^ and the coefficient of variation (CV)^10^^,^^19^ to ensure that the choice of methods had not affected the outcome.

## RESULTS

### Description of data

In 884 people with baseline data: 291 (33%) had CKD at cohort entry, 375 (42%) were diagnosed with CKD on screening and 218 (25%) had only a single test above diagnostic thresholds, and thus did not meet the full KDIGO criteria for CKD. Mean age was 74.3 years (SD 6.7), 54% were female, and the majority of people exhibited either normal renal function (eGFR >60 ml/min/1.73 m^2^ and ACR <3 mg/mmol) (*n* = 296, 33%) or CKD stage 3a (*n* = 279, 32%) at baseline (Table 1).

**Table 1.** Baseline characteristics of the cohort

**Characteristic**	**All participants**	**CKD at baseline**	**Newly diagnosed CKD**	**No CKD^a^**
**Full cohort**				
*n*	884	291	375	218
Age, years, mean (SD)	74.3 (6.7)	75.1 (6.8)	74.1 (6.4)	73.7 (7.1)
Sex, female, %	54	56	50	58
eGFR (ml/min/1.73 m^2^), mean (SD)	63.9 (15.8)	55.9 (13.8)	68.3 (15.3)	67.1 (15.2)
**Stage of CKD, *n* (%)**				
Normal kidney function^b^	296 (33)	70 (24)	135 (36)	91 (42)
1	34 (4)	0	24 (6)	10 (5)
2	114 (13)	17 (6)	77 (21)	20 (9)
3a	279 (32)	120 (41)	93 (25)	66 (30)
3b	88 (10)	60 (21)	18 (5)	10 (5)
4	5 (1)	5 (2)	0	0
Missing	68 (8)	19 (7)	28 (7)	21 (10)

**Included in progression analysis**				
*n*	686	238	268	180
Age, years, mean (SD)	74.0 (6.7)	74.9 (6.6)	73.8 (6.5)	73.1 (7.0)
Female, %	54	55	51	58
eGFR (ml/min/1.73 m^2^), mean (SD)	62.9 (15.3)	55.5 (13.2)	67.2 (15.2)	66.4 (14.4)
**Stage of CKD, *n* (%)**				
Normal kidney function^b^	214 (31)	53 (22)	89 (33)	72 (40)
1	23 (3)	0	16 (6)	7 (4)
2	82 (12)	13 (5)	54 (20)	15 (8)
3a	215 (31)	96 (40)	65 (24)	54 (30)
3b	69 (10)	49 (21)	12 (4)	8 (4)
4	5 (1)	5 (2)	0	0
Missing	78 (11)	22 (9)	32 (12)	24 (13)

a *Participants had one eGFR or ACR suggestive of CKD, but did not meet the full KDIGO/NICE criteria.*

b *Normal kidney function: those with eGFR* >*60 ml/min/1.73 m^2^and ACR* <*3 mg/mmol. ACR = albumin–creatinine ratio. CKD = chronic kidney disease. eGFR = estimated glomerular filtration rate. KDIGO = Kidney Disease Improving Global Outcomes. NICE = National Institute for Health and Care Excellence. SD = standard deviation.*

**Figure 1.**
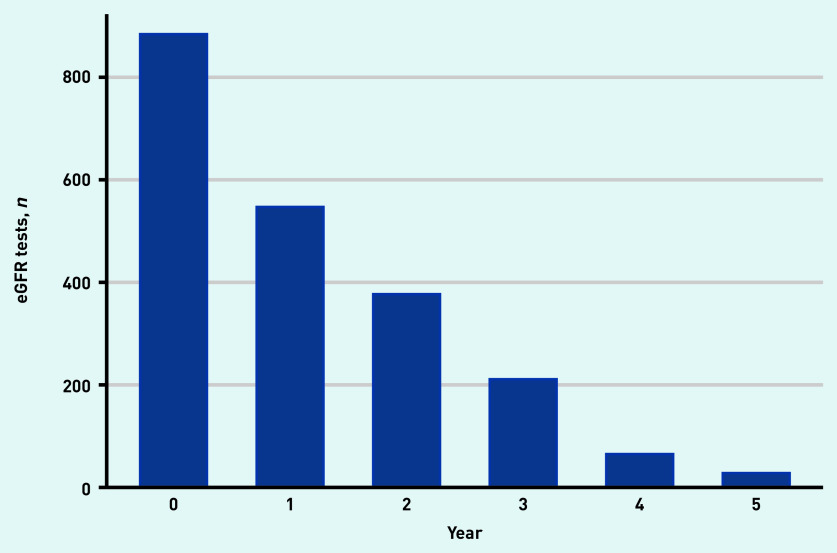
*Number of participants with estimated glomerular filtration (eGFR) tests at their baseline visit and subsequent eGFR tests for each year after entering the cohort.*

**Figure 2.**
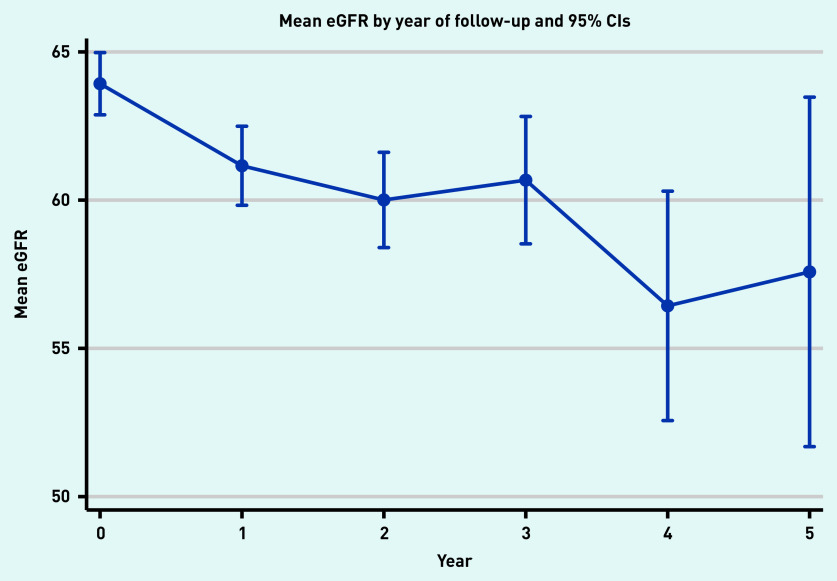
*Mean estimated glomerular filtration (eGFR) for people with up to five eGFR tests (*n *= 884).*

There were 547 people (62%) who had a follow-up eGFR measurement 1 year from baseline; 377 (43%) 2 years from baseline; 211 (24%) 3 years from baseline; 65 (7%) 4 years from baseline; and 28 (3%) with an eGFR 5 years from their baseline visit (Figure 1). Median follow-up time was 2.1 years. Mean eGFR for the population at baseline and years 1, 2, 3, 4, and 5 was 63.9 (standard error [SE] 0.53), 61.4 (SE 0.65), 60.4 (SE 0.79), 60.7 (SE 1.06), 56.6 (SE 1.94); and 57.3 (SE 2.95) ml/min/1.73 m^2^, respectively (Figure 2).

In total, 686 participants had ≥2 eGFR measurements during follow-up and were included in the analysis of CKD progression. Mean age and sex were similar to the full cohort (Table 1). Of these 686 participants, 164 (24%) experienced rapid GFR decline (>5 ml/min/1.73 m^2^ eGFR decrease per year), 27 (4%) experienced progression of >15 ml/min/1.73 m^2^/year, and 48 (7%) experienced a 25% decrease in eGFR combined with progression to the next stage. In total, 30 of 423 (7%) people experienced a sustained decline in the slope who had ≥3 eGFR measurements of >5 ml/min/1.73 m^2^. Additionally, 185 (27%) people experienced improvement in eGFR of at least 5 ml/min/1.73 m^2^/year and 82 of 394 (21%) people who fell into CKD stages 1–4 at the baseline visit experienced remission of their CKD at some point during follow-up. These data and numbers of participants with known CKD, CKD identified by screening, and without CKD who progressed are shown in Table 2.

**Table 2.** Progression of eGFR in participants with >1 eGFR test result

**Participant characteristic**	**All participants, *n* (%)^a^ (*n* = 686)**	**CKD at baseline, *n* (%)^b^ (*n* = 238)**	**Newly diagnosed CKD, *n* (%)^b^ (*n* = 268)**	**No CKD, *n* (%)^b,c^ (*n*= 180)**	**Time to progression, days, mean (SD)**
GFR decline >5 ml/min/1.73 m^2^/year	164 (24)	58 (35)	73 (45)	33 (20)	457 (160)

Progressed >15 ml/min/1.73 m^2^/year	27 (4)	5 (19)	17 (63)	5 (19)	418 (82)

Progressed one CKD stage and 25% decline compared with baseline	48 (7)	21 (44)	20 (42)	7 (15)	543 (275)

eGFR improved by ≥5 ml/min/1.73 m^2^/year	185 (27)	44 (24)	59 (32)	49 (26)	456 (188)

**Of 423 people with ≥3 eGFR measurements**					
Progressed >5 ml/min/1.73 m^2^/year based on regression of ≥3 measurements	30 (7)	9 (30)	17 (57)	4 (13)	428 (188)

**Of 394 people with an eGFR or ACR stage 1–4 at baseline**					
Remission (eGFR>60 ml/min/1.73 m^2^ and ACR <3 mg/mmol)	82 (21)	22 (27)	36 (44)	24 (29)	593 (292)

a *Column percentages.*

b *Row percentages.*

c *Participants with ‘no CKD’ at baseline had one eGFR or ACR suggestive of CKD, but did not meet the full KDIGO/NICE criteria. ACR = albumin–creatinine ratio. CKD = chronic kidney disease. eGFR = estimated glomerular filtration rate. KDIGO = Kidney Disease Improving Global Outcomes. NICE = National Institute for Health and Care Excellence. SD = standard deviation.*

During follow-up, there were 224 withdrawals in the full cohort (*n* = 884) and 27 deaths, and for those with ≥2 eGFR measurements (*n* = 686), there were 122 withdrawals and 17 deaths.

### Regression models

The univariate Cox model revealed significant associations of rapid eGFR decline with being a former smoker, higher systolic and diastolic blood pressure, higher eGFR, and higher log ACR at baseline. In the multivariable analysis, being a former smoker (HR 1.457, 95% CI = 1.033 to 2.054), higher systolic blood pressure (HR 1.013, 95% CI = 1.004 to 1.022), and being known to have CKD at cohort entry (HR 1.670, 95% CI = 1.140 to 2.446) were associated with rapid GFR decline (Table 3). Those with CKD stages 3a or 3b at baseline were less likely to evidence rapid GFR decline, compared with those with normal kidney function. Extending the model to include history of comorbidities at baseline did not identify any other factors to be significantly associated with rapid eGFR decline (see Supplementary Table S2).

**Table 3.** Predictors of rapid decline in eGFR (>5 ml/min/1.73 m^2^/year from baseline) using Cox proportional hazards

**Covariate**	**Univariable analysis, HR (95% CI)**	***P*-value**	**Multivariable analysis, HR (95% CI) (*n* = 568)**	***P*-value**
Age, years	1.005 (0.983 to 1.028)	0.663	1.012 (0.985 to1.040)	0.389

Sex (male compared with female)	0.887 (0.653 to 1.204)	0.443	0.753 (0.518 to 1.096)	0.139

BMI, kg/m^2^	1.025 (0.997 to 1.054)	0.083	1.030 (0.998 to 1.062)	0.068

Smoking status (compared with never smoker)				
Current smoker	0.761 (0.280 to 2.076)	0.594	0.858 (0.304 to 2.424)	0.773
Former smoker	1.388^a^ (1.021 to 1.887)	0.037	1.457^a^ (1.033 to 2.054)	0.032

Alcohol use (compared with no alcohol)	0.833 (0.602 to 1.152)	0.270	0.740 (0.508 to 1.076)	0.115

Level of education (higher education compared with secondary)	1.098 (0.782 to 1.542)	0.590	1.419 (0.953 to 2.112)	0.085

Systolic blood pressure, mg Hg	1.017^a^ (1.009 to 1.025)	<0.0001	1.013^a^ (1.004 to 1.022)	0.006

Diastolic blood pressure, mg Hg	1.014^a^ (1.001 to 1.028)	0.041		

Ethnicity (white compared with black and minority ethnic)	0.582 (0.216 to 1.570)	0.285	0.550 (0.169 to 1.792)	0.321

Waist:hip ratio	1.743 (0.374 to 8.120)	0.479		

Waist circumference	1.010 (0.999 to 1.020)	0.050		

CKD stage at baseline (compared with normal kidney function)^b^				
1	1.695 (0.918 to 3.130)	0.092	1.521 (0.722 to 3.206)	0.270
2	1.191 (0.790 to 1.796)	0.405	0.993 (0.596 to 1.654)	0.987
3a	0.351^a^ (0.229 to 0.540)	<0.0001	0.218^a^ (0.129 to 0.368)	<0.0001
3b	0.332^a^ (0.166 to 0.666)	0.002	0.267^a^ (0.127 to 0.563)	0.001

Log ACR	1.174^a^ (1.033 to 1.334)	0.014	1.116 (0.935 to 1.332)	0.224

Known CKD at baseline (yes versus no)	1.017 (0.741 to 1.394)	0.919	1.670^a^ (1.140 to 2.446)	0.008

a *Statistical significance at 5% (P* ≤ *0.05).*

b *Normal kidney function: those with eGFR* >*60 ml/min/1.73 m^2^and ACR* <*3 mg/mmol. ACR = albumin–creatinine ratio. BMI = body mass index. CKD = chronic kidney disease. eGFR = estimated glomerular filtration rate. HR = hazard ratio.*

There were no associations between variability in eGFR before the baseline visit and rapid eGFR decline. Including eGFR variability in the multivariable analysis resulted in female sex, having higher body mass index (BMI), being a former smoker, and having stage 3a CKD at baseline being significantly associated with progression of CKD. Results did not change substantially when variability was defined as mean and SD or CV of eGFR in place of VIM (see Supplementary Table S3).

Lower systolic blood pressure and lower log ACR were significantly associated with CKD improvement in the univariable analysis. Only lower systolic blood pressure remained significant in the multivariable analysis (see Supplementary Table S4). Those with lower ACR were more likely to experience remission and those with known CKD were less likely to experience remission in the univariable analysis. Only lower ACR remained significant in the multivariable analysis (see Supplementary Table S4).

**Figure 3.**
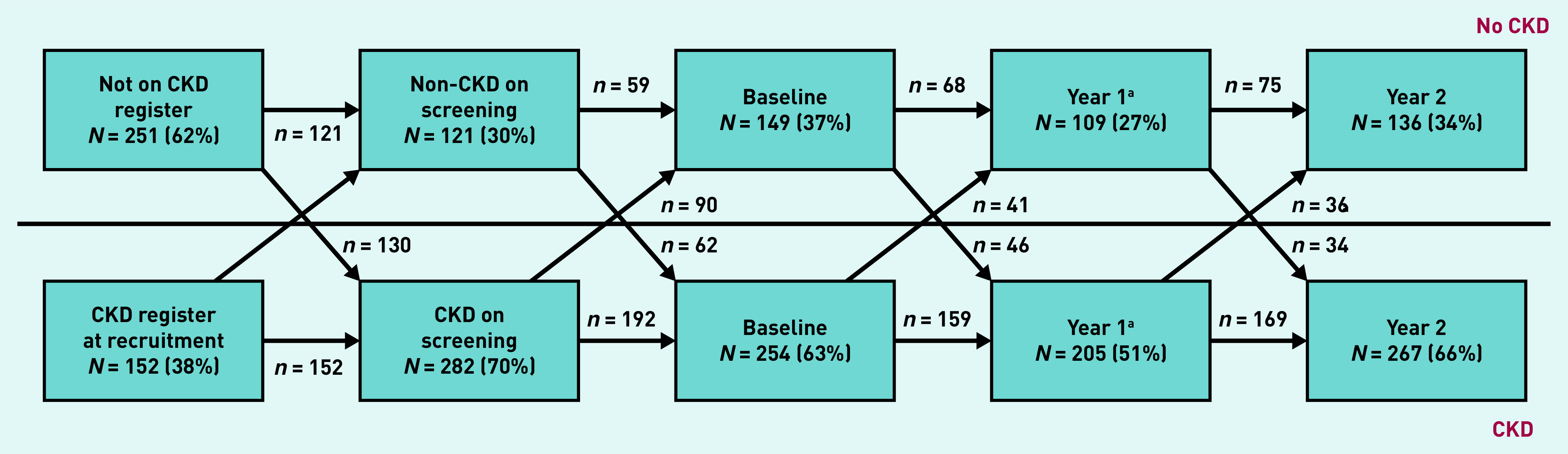
***Flowchart showing people moving across the diagnostic threshold for chronic kidney disease (CKD) for those with complete baseline and year 2 follow-up (*n *= 403). There were 139 participants (55%) with CKD at baseline who remained below the CKD threshold throughout follow-up at both year 1 and year 2.*** *^a^****Year 1: data for 89 participants missing.***

Figure 3 shows that people move across the CKD diagnostic threshold over a 2-year follow-up in both directions, with 30%–40% of the cohort falling below the eGFR/ACR threshold for CKD at any one time and 139 participants remaining below CKD diagnostic thresholds over the entire follow-up. When CKD stage changes between baseline and year 2 were mapped, it was found that, although 59% of people remained in the same stage, 41% had changed stage (see Supplementary Table S5). Of participants who were classified as having CKD at baseline, 83% were also classified as having CKD at their 2-year follow-up, but 17% fell into the non-CKD range after 2 years (see Supplementary Table S6). Of those with CKD at baseline, 8% of people moved to a less advanced CKD stage and 18% moved to a more advanced CKD stage after 2 years.

## DISCUSSION

### Summary

In this older primary care population with early-stage CKD or some renal impairment, the current study determined that 24% of people experienced rapid eGFR decline of at least 5 ml/min/1.73 m^2^/year. It was also identified that having a higher BMI, being a smoker, having a higher systolic blood pressure, and being known to have CKD at cohort entry were all associated with rapid GFR decline in this population. In addition to eGFR and CKD progression, it was found that 27% of people experienced an improvement in eGFR, and that having a lower systolic blood pressure showed the strongest association with this improvement. Those who experienced remission were most likely to have a lower ACR. During 2 years of follow-up, at any one time, approximately 30% of people were in remission, despite many of them meeting the definition of CKD at some point.

### Strengths and limitations

This article provides reliable, prospectively collected data from an observational study that will help clinicians to prioritise those at greatest need of closer monitoring or medication reviews. Although eGFR decline is indicative of deterioration in kidney function and CKD, the data in the current study demonstrate that, in some instances, it may be a temporary decrease, such as an episode of acute kidney injury following which kidney function recovers. This and other potential reasons for a temporary decline in kidney function include biological and analytical variability, which are outlined in Supplementary Table S7. The KDIGO guidelines^1^ state that confidence in the precision of the decline increases with an increasing number of serum creatinine tests and duration of follow-up. The impact of a larger number of eGFR results on this analysis were explored, but fewer events meant that it was not possible to conduct a full analysis. Furthermore, those with a more advanced stage of CKD at baseline were less likely to experience eGFR progression, which represent ‘regression to the mean’. This has been noted in other studies,^22^^,^^23^ and arises when day-to-day biological variations and imprecision in the measurement result in some people by chance having a spuriously high eGFR at their baseline assessment, meaning they may be more likely to meet the definition of progression in subsequent measurements. In the current study, therefore, numbers of participants who meet different definitions of progression and sustained progression were also reported. In addition, the numbers of people whose eGFR improved and the number who experienced remission, of those who met the definition of having CKD, have been reported.

The follow-up time for this cohort was relatively short, which may have had an impact on the numbers of people who experienced progression. Whereas the proportion of participants experiencing a short-term rapid decline in GFR was relatively high (24%), the proportion with GFR decline sufficient to prompt referral to secondary care was low (7%).

This analysis has found that those who were already known to have CKD may be more likely to experience rapid eGFR decline than those who were identified to have CKD on screening but, depending on the statistical method used, this was not consistent, which may result from a lack of statistical power. However, characteristics of the OxRen baseline population have already been reported and those with existing CKD had lower eGFR than those who were diagnosed through screening,^15^ so may have been living longer with CKD.

### Comparison with existing literature

A previous study reported that 18% of people experienced CKD progression over 5 years when defining progression as a 25% decline in eGFR combined with moving to a higher CKD stage.^11^ Using this definition, it was found that only 7% of this cohort progressed over a shorter follow-up time. Furthermore, if the slope of ≥3 eGFR measurements was used to define progression, only 7% of the OxRen cohort progressed, suggesting that some of those with rapid decline may have had more variability in their eGFR measurements. Other studies reported progression in 15% to 20% of participants,^24^^,^^25^ with higher rates of progression in those with the most severe CKD.^24^ The proportion who experienced an improvement in eGFR was similar to other studies, ranging from 12% to 27% depending on stage of CKD.^11^^,^^24^^,^^25^

The current study found that kidney function fluctuated during follow-up as people move back and forth across the 60 ml/min/1.73 m^2^ eGFR threshold over time and, therefore, that a substantial minority of participants evidenced ‘remission’ of their CKD. This type of variability in eGFR has been described previously,^24^^,^^25^ with one study in UK primary care reporting a similar observation,^11^ such that over 25% of participants were ‘in remission’ at any one time. A slightly higher proportion (30%–40%) demonstrating remission after 1 and 2 years of follow-up was found in the current study, but some people in the cohort had an earlier stage of CKD, or did not fully meet the definition for CKD on cohort entry. Many of these fluctuations will result from normal biological variability and measurement error in people who were near measurement thresholds. However, those who remain in remission at baseline and year 1 have lower mortality (3.2%) compared with those who meet criteria for CKD at both visits (15.7%).^11^ The issue of CKD ‘remission’ and the associated clinical implications warrants further investigation.

However, regardless of individuals who cycle back and forth between CKD and ‘remission’, the overall outcome during 2 years of follow-up was an overall trend towards a lower eGFR over a 2-year period. Whether those with rapid GFR decline in this study go on to develop adverse health outcomes is yet to be determined. With continued follow-up of this cohort, data on long-term health and mortality can be evaluated in relation to CKD progression.

In the current analysis of variability in eGFR before entering the cohort, no association with subsequent eGFR decline was found. To the authors’ knowledge, only one previous study has used similar methods to explore eGFR variability and found that it was associated with increased mortality in a population with existing risk factors for cardiovascular disease.^10^

### Implications for practice

This work adds to existing knowledge of CKD progression and GFR decline as well as tools that can be used to identify risk of progression. The findings will help clinicians identify patients who may be at greatest risk of deteriorating renal function in order to prioritise closer monitoring or medication reviews to reduce cardiovascular risk including blood pressure management and lifestyle interventions.^1^ Particular attention should perhaps be directed towards females who are overweight with a history of smoking. Nevertheless, it should also be appreciated that GFR may fluctuate over time and that only a small proportion of people with a sustained and large GFR decline warrant referral to a nephrology service.

In the OxRen cohort, those known to have CKD on cohort entry were more likely to experience rapid GFR decline than those who were only found to have CKD on screening and those who did not meet the KDIGO criteria for CKD at their baseline assessment. This preliminary analysis casts some doubt on the value of screening for CKD in older populations as those at highest risk may be those already known to have CKD.

In conclusion, this work has improved understanding about how eGFR fluctuates over time and will help clinicians identify those who may benefit most from closer monitoring and appropriate treatment to minimise risks of adverse outcomes.
